# Coixol Protects Against Acute Kidney Injury by Reducing Cell Senescence

**DOI:** 10.3390/biology14050560

**Published:** 2025-05-17

**Authors:** Kang Li, Xiaoxue Wang, Huidi Tang, Feifan Wang, Zetong Qu, Xiaojie Wang, Xiang Li, Xiaoqing Cao

**Affiliations:** 1 Department of Pharmacology, School of Basic Medical Sciences, Shandong University, Jinan 250012, China; 202100412082@mail.sdu.edu.cn (K.L.); wangxiaoxuejn@126.com (X.W.); 202100260055@mail.sdu.edu.cn (H.T.); 202100412096@mail.sdu.edu.cn (F.W.); 202123411023@mail.sdu.edu.cn (Z.Q.); wangxiaojie@sdu.edu.cn (X.W.); 2 Department of Pharmacy, Shandong Public Health Clinical Center, Shandong University, Jinan 250013, China; 3 Department of Cardiology, Shandong Public Health Clinical Center, Shandong University, Jinan 250013, China

**Keywords:** acute kidney injury, Coixol, cellular senescence, Plaur, machine learning, molecular docking, molecular dynamics

## Abstract

Acute kidney injury (AKI) is associated with increased in-hospital mortality, yet effective therapeutic agents remain limited. Coixol, a polyphenolic compound derived from Coix, possesses anti-inflammatory properties, but its role in AKI remains unclear. In this study, we demonstrate that Coixol exerts protective effects against ischemia/reperfusion (I/R)-induced AKI by alleviating cellular senescence. Coixol treatment significantly reduced serum creatinine (SCr) and blood urea nitrogen (BUN) levels and decreased the expression of KIM1 and NGAL. RNA sequencing and validation experiments revealed that Coixol suppressed cellular senescence in AKI. Through a weighted gene co-expression network analysis and machine learning, we identified Plaur as a key target of Coixol, which was further validated using RNA-seq data. Notably, Plaur overexpression in AKI mice diminished the protective effects of Coixol, confirming its crucial role. Additionally, molecular docking and molecular dynamics simulations demonstrated strong binding affinity between Coixol and Plaur. These findings highlight Coixol as a promising renal protective agent targeting Plaur and cellular senescence in AKI.

## Simple Summary

Acute kidney injury is a serious condition that can cause kidney failure and death, but there are few effective treatments available. In this study, we explored the protective effects of a natural compound called Coixol, which is found in a plant called Coix. We found that Coixol can significantly reduce kidney damage caused by restricted blood flow, a common cause of acute kidney injury. Coixol exerts its protective effect by helping to slow the aging process of kidney cells, which normally accelerates during injury and worsens the damage. We also discovered that a specific protein, called Plaur, plays a key role in this process, and Coixol can lower the levels of this harmful protein. Further studies showed that Coixol binds tightly to Plaur, helping to block its harmful effects. These findings suggest that Coixol may be a promising new treatment to protect the kidneys and could one day help patients recover faster and more completely after kidney injury. This research opens the door for new approaches to treating a condition that currently has very few options.

## 1. Introduction

Acute kidney injury (AKI), a complex syndrome defined by a rapid decline in renal function [1,2,3], affects 15% of hospitalized patients and 40–60% of intensive care unit patients [4]. Risk factors for AKI include severe infection, hypovolemia, major trauma, and nephrotoxic dru42gs 1. Although numerous pharmacological agents have shown efficacy in animal models, such as leonurine’s effectiveness in mitigating cisplatin-induced AKI [5] and nuciferine’s effectiveness in alleviating folic acid-induced AKI [6], their clinical translation remains challenging. Clinical trials have failed to demonstrate a safe therapeutic agent for patients with AKI. Thus, there is an urgent need for high-efficacy, cost-effective, and low-adverse-reaction therapeutic strategies for AKI.

Coixol (6-methoxy-2(3H)-benzoxazolone), an alkaloid from scoparia dulcis, belongs to the class of organic compounds known as benzoxazolones that exert protective effects on diabetic kidney diseases [7], suggesting that Coixol may be an effective novel agent for treating kidney diseases. Mechanically, Coixol plays a crucial role in regulating insulin secretion [8] and suppressing inflammatory responses by inhibiting the activation of the Toll-like receptor 4 signaling pathway during infection [9,10]. In an experimental study on Toxoplasma infection, Coixol inhibited the inflammatory cascade by regulating the HSP70/TLR4/NF-κB pathway in Kupffer cells [11]. Moreover, Coixol could suppress NF-κB, MAPK pathways, and NLRP3 inflammasome activation in lipopolysaccharide-induced RAW 264.7 cells. It is worth noting that Coixol could be used not only in in vitro studies but also in animal studies without showing toxic effects [8].

Cellular senescence represents a typical irreversible and stable proliferation arrest state associated with functionality, structure, and morphology alterations [12]. Senescent cells undergo significant alterations, including metabolic reprogramming, autophagic regulation, and the secretion of a heterogenous bioactive molecular profile known as the Senescence-Associated Secretory Phenotype (SASP), which can influence adjacent cells and potentially lead to chronic inflammation [13]. The SASP includes interleukin6 (IL6), transforming growth factor-α (TNF-α), monocyte chemoattractant protein1 (MCP1), and others, exerting its effects through paracrine and autocrine signaling [14,15]. Furthermore, senescent cells express senescence-associated β-galactosidase (SA-β-gal), and both SASP and SA-β-gal serve as biomarkers of cellular senescence [16]. Notably, cellular senescence has been revealed to be one of the most important mechanisms involved in AKI [17,18]. Various stimuli in AKI can lead to DNA damage, mitochondrial dysfunction, and increased reactive oxygen species (ROS), all of which are common causes of cellular senescence [19]. During AKI, both the renal cortex and medulla can undergo senescence, affecting tubular epithelial cells, podocytes, and vascular smooth muscle cells 14. Among them, tubular epithelial cell senescence is an early event that contributes to the accumulation of senescent cells following kidney injury, thereby promoting disease progression [20]. Enhancing tubular epithelial cell repair may help mitigate AKI [21]. A recent study showed that the elimination of senescent cells can promote renal regeneration after AKI and extend lifespan [22]. Therefore, cellular senescence plays a critical role in AKI.

In this study, we discovered a previously unrecognized role of Coixol in mitigating renal injury in AKI, specifically by reducing cellular senescence in tubular cells, suggesting that Coixol may be a novel senotherapeutic agent.

## 2. Materials and Methods

### 2.1. Animal Studies

All experimental protocols were approved by the Institutional Animal Care and Use Committee of the School of Basic Medical Sciences, Shandong University, and conducted in accordance with the National Institutes of Health Guide for the Care and Use of Laboratory Animals. The animals were randomly assigned to different experimental groups using a computer-generated randomization schedule. The investigators who performed the surgeries and those who assessed the outcomes were blinded to the group assignments to minimize bias. Kidney ischemia–reperfusion was performed under standardized conditions at 24 ± 0.5 °C, with the average intraabdominal temperature being maintained at 35 ± 2 °C during the operating period using a heating pad controlled by rectal temperature [23,24]. The renal pedicle was clamped for 35 min. For survival experiments, animals were observed for 1 day after all surviving animals were free of signs of illness. Coixol was treated through intraperitoneal injection in PBS at a dosage of 20 mg/kg. Normal control mice were administered the same amount of PBS. After 1 day, the mice were sacrificed, and tissue samples were collected for further analysis. The blood samples of the normal (n = 4), AKI (n = 3) and Coixol groups (n = 3) were submitted for RNA sequencing.

To determine gene overexpression in the animals, all mice were administered the uPAR expression plasmid (pFlag-plaur) or control plasmid by rapidly injecting a large volume of DNA solution through the tail vein [25].

### 2.2. Serum Creatinine (SCr) and Blood Urea Nitrogen (BUN)

SCr and BUN were measured from 30 μL of whole blood on an automated analyzer (Beckman Coulter, Brea, CA, USA) with a fast test strip [26].

### 2.3. RT-qPCR

Total RNA was isolated from tissues or cells using Trizol reagent (Invitrogen, Carlsbad, CA). The mRNA levels for the target genes were analyzed using real-time quantitative RT-PCR using a Bio-Rad iCycler system (Bio-Rad, Hercules, CA, USA). The levels of the housekeeping gene β-actin were used as an internal control. The specific primers used in this study are listed in Supplemental Table S1.

### 2.4. SA-β-Gal Staining

SA-β-gal staining was performed according to the manufacturer’s instructions by using its staining kit [27]. Briefly, frozen kidney tissue sections (5 μm thick) or HPC growing on slides were washed twice with PBS and fixed with a fixative solution for 15 min at room temperature after the corresponding treatments. Then, the kidney tissue sections were stained with 1 mL of β-galactosidase staining solution per well overnight at 37 °C. After being cultured at 37 °C without CO_2_, the sections or slides were washed twice with PBS, and pictures were taken under a light microscope. Ten randomly chosen microscopic fields were imaged and quantified by blindly counting the positive cells per chamber.

### 2.5. Immunofluorescence Staining

The tissues were transferred to 4% PFA and fixed at 4 °C overnight. Then, they were paraffin-embedded and cross-sectioned (4 μm) for immunofluorescence staining. Images were obtained using an LSM980 laser scanning confocal microscope (ZEISS, Oberkochen, Germany) system equipped with a Plan-Apochromat 63 ×/ 1.4 objective. The tissue sections were incubated with different primary antibodies and subsequently incubated with secondary Alexa 488 or 594 conjugated antibody (Abcam). Nuclei were counterstained with DAPI (Roche, Mannheim, Germany). The antibody used in this study is shown in Supplemental Table S2.

### 2.6. Identifying Key Pathways of AKI

The limma package was used to analyze the results of RNA sequencing, defining genes with a *p-*value < 0.05 and |logFC| ≥ 0.5 as DEGs. A volcano plot of the DEGs and a PCA plot of the samples were generated using the ggplot2 and scatterplot3d packages, respectively. The DEGs between AKI and normal kidneys were subjected to a KEGG enrichment analysis using the clusterProfiler package, and visualization was performed using the ggplot2 package. A KEGG pathway analysis not only facilitates annotating the function of the genes themselves but also annotating genes involved in various signaling pathways. A *p-*value < 0.05 and gene counts ≥ 5 were considered statistically significant.

### 2.7. Data Sources

The search formula “(“acute kidney injury”[MeSH Terms] OR acute kidney injury[All Fields]) AND “Homo sapiens”[porgn] AND (“Expression profiling by array”[Filter] AND “attribute name tissue”[Filter])” was used to select relevant gene expression datasets from the GEO Database (http://www.ncbi.nlm.nih.gov/geo (accessed on 27 September 2024)). The GSE30718 dataset was finally selected and downloaded from the GPL570 platform ([HG-U133_Plus_2] Affymetrix Human Genome U133 Plus 2.0 Array), which collected 28 AKI samples and 11 normal tissue samples. The cellular senescence-related gene set was downloaded from the GeneCards database (https://www.genecards.org/ (accessed on 27 September 2024)). Only genes with a relevance score greater than 20 were included for subsequent analysis [28].

### 2.8. Identifying Key Cellular Senescence-Related Genes

The limma package was employed to analyze GSE30718, defining genes with *p-*value < 0.05 and |logFC| ≥ 0.5 as DEGs. Volcano plots of the DEGs were generated using the ggplot2 package. Subsequently, a principal component analysis (PCA) of GSE30718 was performed using the scatterplot3d and ggplot2 packages to visualize sample grouping. Using the WGCNA package, a gene co-expression network was constructed to investigate the relationship between genes and phenotypes. Initially, the top 25% of genes with the highest variance were selected for subsequent analysis. The soft threshold parameter was set to a power of 17, and a scale-free R^2^ value of 0.9 was used for the weighted adjacency matrix, which was further transformed into a topological overlap matrix (TOM). Additionally, “TOMType” was set to “unsigned”. By setting the “minModuleSize” to 30, modules were obtained using the TOM-based dissimilarity metric (1-TOM) based on the hierarchical clustering tree algorithm. Each module was assigned a random color. After analyzing the 16 gene modules obtained, the black, cyan, and yellow modules were selected for further analysis.

### 2.9. Using Machine Learning for Screening Hub Genes and Validation

To further filter the optimal diagnostic genes for AKI, Least Absolute Shrinkage and Selection Operator (LASSO) and random forest (RF) were adopted. A LASSO logistic regression analysis is a regression methodology proficient at selecting variables to enhance predictive accuracy. It serves as a technique for both regression and variable selection, employing regularization to improve predictive accuracy and enhance the comprehensibility of statistical models. RF is an ensemble prediction method capable of managing a vast number of input variables while assessing their significance. We employed the glmnet and randomForest packages to conduct LASSO and RF analyses. RNA sequencing data were used as validation datasets. The box plots were constructed using the ggplot2 package to assess the expression levels of the hub genes.

### 2.10. Molecular Docking

The protein crystal structure of Plaur (PDBID = 6AEX) used for docking was obtained from the PDB database (https://www.rcsb.org/ (accessed on 10 January 2025)). The 3D structure of the small molecule Coixol was constructed using Chem3D, and its energy was minimized under the MMFF94 force field. Molecular docking was performed using AutoDock Vina software [29]. Prior to docking, the receptor protein was prepared using PyMol, including the removal of water molecules, salt ions, and small molecules. The docking box was set with its center at the centroid of the ligand in the original crystal structure, and the box size was 25 × 25 × 25 Å^3^. During docking, the global search exhaustiveness was set to 32, with other parameters kept at their default settings. The docking pose with the highest docking score was considered the binding conformation, and the docking results were visualized using PyMol. In this study, a binding energy of <−5.0 kJ/mol indicates strong binding activity [30]. Additionally, Li’s research identified PLAUR as a potential target gene of urokinase and performed molecular docking. This study used it as a positive control for comparison [31].

### 2.11. Molecular Dynamics (MD) Simulation

The small molecule–protein complex obtained from docking was used as the initial structure for all-atom MD simulations, which were performed using the AMBER 22 software [32]. The small molecule and protein were described using the GAFF2 force field for small molecules and the ff14SB force field for proteins, respectively [33,34]. Prior to the simulation, energy optimization was performed, including 2500 steps of the steepest descent method and 2500 steps of the conjugate gradient method. After energy optimization, the system was heated for 200 ps at a constant volume and with a gradual temperature increase from 0 K to 298.15 K. Following this, a 500 ps NVT (constant number of particles, volume, and temperature) ensemble simulation was performed to allow solvent molecules to further equilibrate within the solvent box. Finally, a 500 ps NPT (constant number of particles, pressure, and temperature) equilibration simulation was conducted. The complex system was then simulated for 100 ns under NPT conditions with periodic boundary conditions. During the simulation, the nonbonded cutoff distance was set to 10 Å, the Particle Mesh Ewald (PME) method was used to calculate long-range electrostatic interactions, the SHAKE algorithm was applied to constrain hydrogen bond lengths, and the Langevin algorithm was used for temperature control, with the collision frequency γ set to 2 ps^−1^ [35,36,37]. The system pressure was maintained at 1 atm, the integration step size was 2 fs, and the trajectory was saved every 10 ps for subsequent analysis.

### 2.12. Molecular Mechanics Generalized Born Surface Area (MM-GBSA) Studies

The binding free energy between the protein and ligand for all systems was calculated using the MM-GBSA method [38,39,40]. In this study, 90–100 ns of MD trajectory data were used for the calculation, and the specific formula is as follows:

(1) $${\Delta G}_{bind}={\Delta E}_{internal}+{\Delta E}_{VDW}+{\Delta E}_{elec}{+\Delta G}_{GB}+{\Delta G}_{SA}$$

In Equation (1), ΔE_internal_ represents the internal energy, ΔE_VDW_ denotes the van der Waals interaction, and ΔE_elec_ indicates the electrostatic interaction. The internal energy includes the bond energy (E_bond_), angle energy (E_angle_), and torsional energy (E_torsion_). ΔG_GB_ and ΔG_GA_ are collectively referred to as the solvation free energy. G_GB_ represents the polar solvation free energy, and G_SA_ represents the nonpolar solvation free energy. The GB model developed by Nguyen et al. [41] was used to calculate the ΔG_GB_ value (igb = 2). The nonpolar solvation free energy (ΔG_SA_) was calculated based on the product of surface tension (γ) and solvent-accessible surface area (SA) (ΔG_SA_ = 0.0072 × ΔSASA) [42]. Entropy change was neglected in this study due to its high computational cost and low precision [38].

### 2.13. Quantification and Statistical Analysis

The data are expressed as means ± SEM. Statistical analyses were performed with GraphPad Prism (version 8.0, GraphPad Software, San Diego, CA, USA). Bioinformatics analyses were performed with R software (version 4.3.2, New Zealand). The normality assumption of the data distribution was assessed using the Kolmogorov–Smirnov test. Comparisons between the two groups were performed using a two-tailed Student’s *t*-test for normally distributed data and the Mann–Whitney rank sum test for non-normally distributed data. Differences between multiple groups with one variable were determined using a one-way ANOVA followed by Tukey’s post hoc test. To compare multiple groups with more than one variable, a two-way ANOVA was used, followed by Tukey’s post hoc test. All statistical details regarding the *p* value and n can be found in the figure legends. Mice were allocated to different groups in a randomized manner, and the investigators were blinded to this process when performing surgeries and outcome evaluations. The exclusion criteria were based on animal well-being at the beginning of the study. The sample size in each study was based on experience with previous studies in our lab. Slides stained with Sirius red, PAS and immunofluorescence were observed at high magnification fields from randomly selected fields. Each section contained 10 fields. Quantification of injury area was assessed by the Image Pro plus software (version 6.0, USA). The injury score was assessed by tubular dilation, hyaline casts, and detached epithelial cells in tubular lumens as well as detached brush borders. The percentages of tubular injury in each image were calculated by three experienced researchers who were blinded through Adobe Photoshop 2023 software (version 24.1.0, USA).

## 3. Results

### 3.1. Coixol Treatment Significantly Ameliorated IRI-Induced Kidney Injury

**Figure 1:**
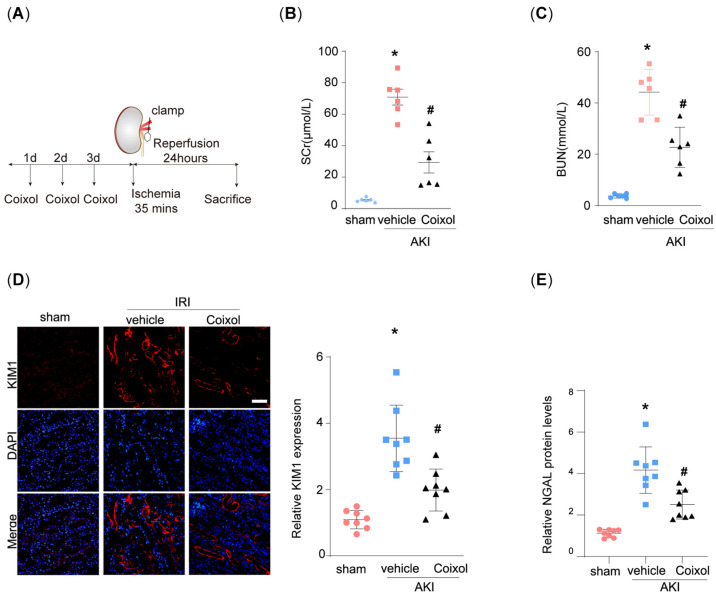
Coixol treatment significantly ameliorated ischemia–reperfusion injury (IRI)-induced acute kidney injury (AKI). (**A**) A schematic graph showing the construction of the mouse model and the experimental design. Three days, two days, and one day before surgery, the mice were intraperitoneally injected with 20 mg/kg of Coixol, respectively. On the day of surgery, renal vessels were clamped for 35 min, followed by 24 h reperfusion. Finally, the mice were euthanized for subsequent experiments. (**B**) Serum creatinine (SCr) levels in different groups of mice (n = 6). (**C**) Blood urine nitrogen (BUN) levels in different groups of mice (n = 6). (**D**) Representative immunofluorescence (IF) images of KIM1 in different groups of mice. Scale bar: 20 μm. (**E**) An ELISA analysis of NGAL protein levels in different groups of mice. (n = 8). All data are expressed as means ± SEM. * *p* < 0.05 vs. sham; # *p* < 0.05 vs. AKI mice.

Firstly, we established the ischemia/reperfusion-induced AKI mouse model (Figure 1A). The physical and biochemical parameters of the AKI mice are shown in Supplementary Table S3. We found that 35 min of ischemia significantly elevated the SCr and BUN levels of model mice (Figure 1B,C). Notably, pretreatment using Coixol significantly ameliorated IRI-induced renal dysfunction. Moreover, we observed that IRI markedly upregulated the expression of renal injury markers KIM1 and NGAL in mice (Figure 1D,E). Notably, pretreatment with Coixol significantly reduced the expression levels of both KIM1 and NGAL compared to untreated IRI mice, indicating its protective effect against IRI-induced renal injury (Figure 1D,E).

### 3.2. Cellular Senescence Served as the Major Signaling Pathway Targeted by Coixol in AKI

**Figure 2:**
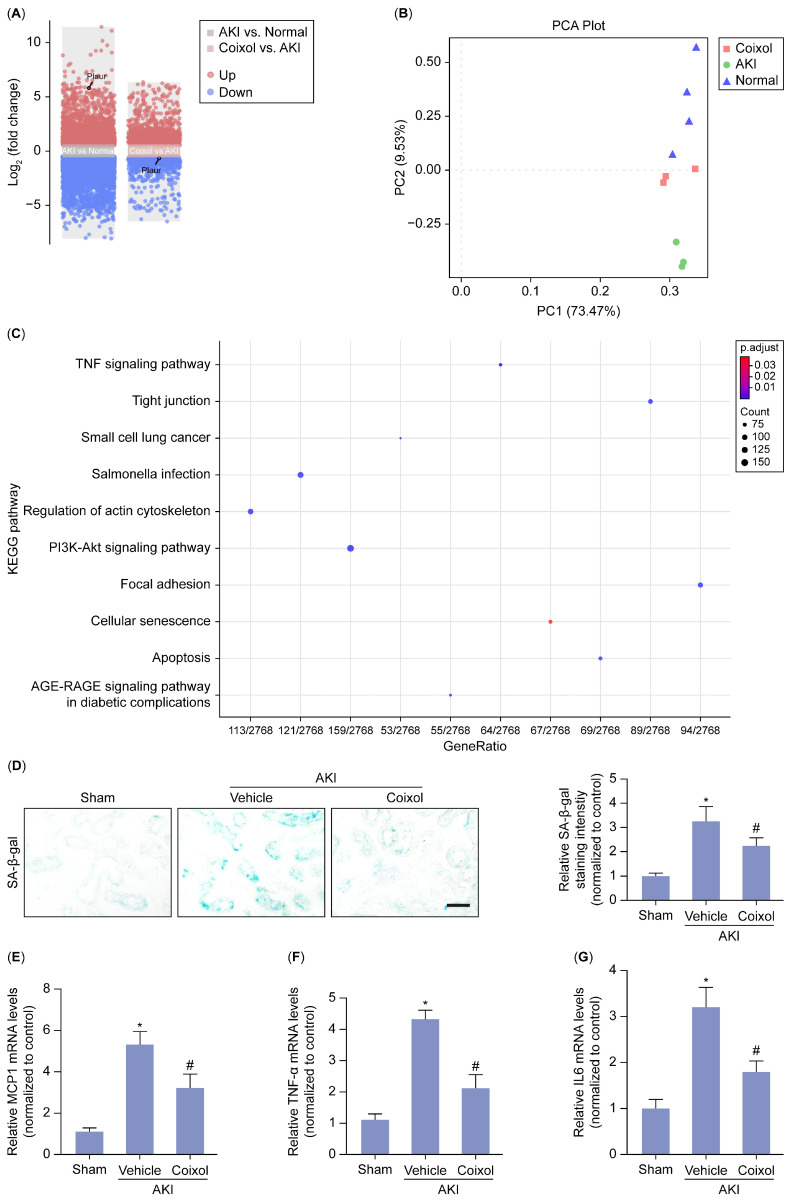
Cellular senescence served as the major signaling pathway targeted by Coixol in AKI. (**A**) A volcano plot of DEGs in group 1 (Coixol vs. AKI) and group 2 (AKI vs. normal tissue). Red and blue represent DEGs with significantly higher and lower expression levels, respectively. (**B**) A PCA plot of samples for RNA-seq. (**C**) The KEGG analysis results of the DEGs for all enriched KEGG terms. (**D**) Representative images and the quantification of SA-β-gal staining levels in different groups of mice. Scale bar: 20 μm. (**E**) The relative mRNA levels of MCP1 in different groups of mice (n = 6). (**F**) The relative mRNA levels of TNF-α in different groups of mice (n = 6). (**G**) The relative mRNA levels of IL-6 in different groups of mice (n = 6). All data are expressed as means ± SEM. * *p* < 0.05 vs. sham; # *p* < 0.05 vs. AKI mice.

To further dissect the mechanism underlying the protection provided by the Coixol treatment in AKI, we performed RNA sequencing on renal tissue from various groups. After analyzing the RNA sequencing results, 7292 differently expressed genes (DEGs) which comprised 3403 upregulated genes and 3889 downregulated genes between the AKI and control groups and 1512 DEGs, which comprised 861 upregulated genes and 651 downregulated genes between the Coixol and AKI groups, were identified. Figure 2A,B depict the volcano plot of the DEGs and the PCA plot of the samples, respectively. A KEGG enrichment analysis revealed the important roles of DEGs in the cellular senescence pathway (Figure 2C) (Supplemental Table S4). Notably, we found that tubular cellular senescence is significantly increased in AKI mice, while Coixol pretreatment ameliorated IRI-induced cellular senescence, as evidenced by the staining intensity of SA-β-gal (Figure 2D). Moreover, SASP is a common feature of cellular senescence and aging. Using an mRNA analysis, we found that Coixol pretreatment ameliorated SASP in AKI mice (Figure 2E–G), suggesting that Coixol may protect against AKI by targeting cellular senescence.

### 3.3. Plaur Was Identified as the Key Cellular Senescence-Related Gene Associated with the Protective Effect of Coixol

**Figure 3:**
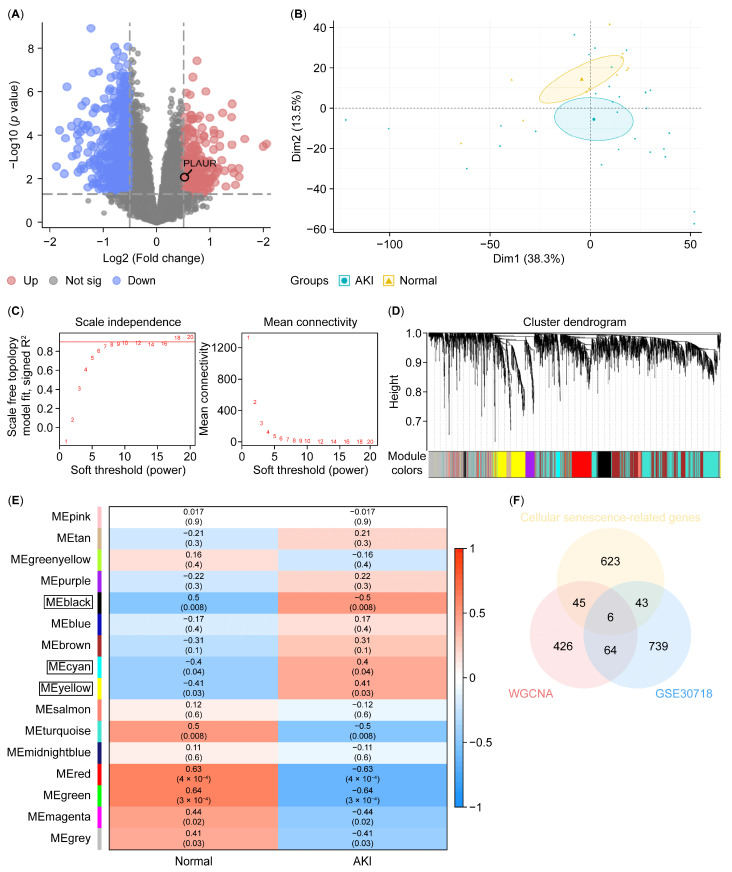
Identifying key cellular senescence-related genes in AKI. (**A**) A volcano plot of DEGs in GSE30718. Red and blue represent DEGs with significantly higher and lower expression levels in AKI groups, respectively. (**B**) A PCA showing different groups. Blue indicates AKI, and yellow indicates normal tissues. (**C**) β = 17 is selected as the soft threshold with the combined analysis of scale independence and mean connectivity. (**D**) A gene hierarchy tree-clustering diagram. Different colors represent different module genes. (**E**) A heatmap of the correlation between module genes and phenotypes. Black, cyan, and yellow modules are chosen. (**F**) A Venn diagram of the module genes, DEGs of GSE30718, and cellular senescence-related genes.

**Figure 4:**
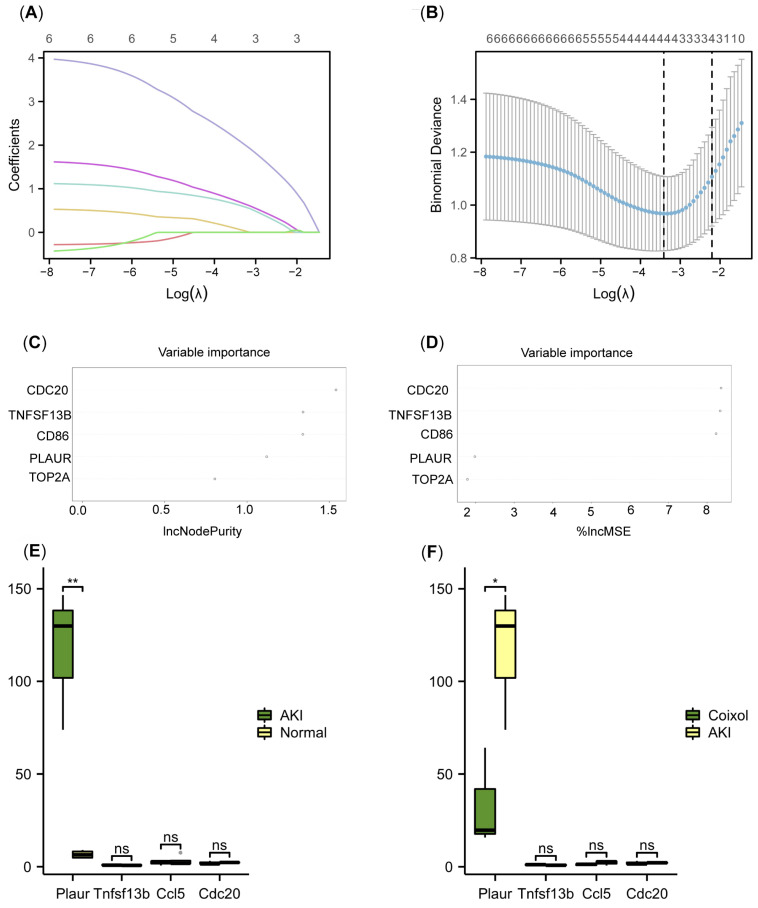
Plaur was identified as the key cellular senescence-related gene associated with the protective effect of Coixol. (**A**,**B**) A LASSO regression algorithm. Each color represents a gene. The best diagnosis model includes TNFSF13B, CCL5, CDC20, and PLAUR. (**C**,**D**) The top 5 genes with variable importance in the RF analysis. According to IncNodePurity and %IncMSE, the order of variable importance was CDC20, TNFSF13B, CD86, PLAUR, and TOP2A. (**E**) The expression of diagnostic markers in AKI samples compared to normal samples. **, *p* < 0.01, Student’s *t*-test. (**F**) The expression of diagnostic markers in Coixol samples compared to AKI samples. *, *p* < 0.05, Student’s *t*-test.

According to our RNA-seq data, Plaur is upregulated in AKI mice, and the Coixol treatment significantly reduced its expression (Figure 2A). Next, we analyzed published RNA sequencing data from the GSE30718 dataset (molecular phenotypes in renal tissue from patients with AKI and normal controls) to verify the results. Figure 3A depicts a volcano plot of the DEGs in human renal tissue from AKI patients and control subjects. The PCA plot demonstrates a distinct separation between AKI samples and normal samples (Figure 3B). WGCNA was applied to identify modules most strongly associated with AKI. We chose β = 17 (scale-free R^2^ = 0.9) based on scale independence and mean connectivity (Figure 3C). A total of 16 co-expression gene modules (GCMs) were generated (including PLAUR), distinguished by different colors (Figure 3D). The correlation between AKI and GCMs was calculated (Figure 3E), with the black, cyan, and yellow modules being selected for subsequent analysis. The intersection genes (6 genes) of module genes, DEGs, and cellular senescence-related genes were obtained (Figure 3F). Furthermore, we applied a machine learning analysis, including LASSO and RF, to analyze the DEGs in AKI. Using LASSO, 4 genes (TNFSF13B, CCL5, CDC20, and PLAUR) were identified from the set of 6 cellular senescence-related genes (Figure 4A,B). According to IncNodePurity and %IncMSE, the order of variable importance was CDC20, TNFSF13B, CD86, PLAUR, and TOP2A (Figure 4C,D). However, in our RNA-seq data, Plaur was the only elevated gene in renal samples from AKI mice compared with normal samples (*p* < 0.05) (Figure 4E), and it was further reduced by Coixol treatment (*p* < 0.05) (Figure 4F), suggesting that Plaur may be the crucial target gene of Coixol that affects cellular senescence in AKI.

### 3.4. Plaur Overexpression Diminished the Protective Effect of Coixol on AKI

**Figure 5:**
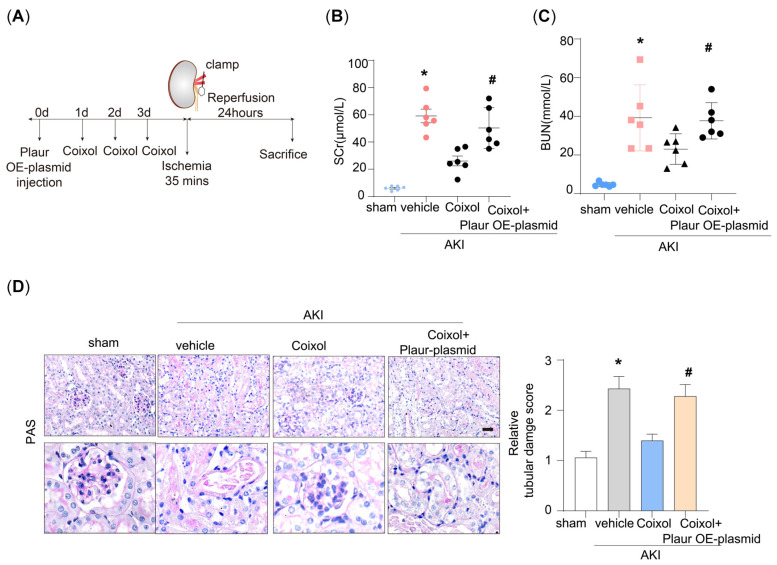
Plaur upregulation diminished the protective effect of Coixol on AKI. **(A**) A schematic graph showing the construction of the mouse model. First, the overexpression of the uPAR protein in mice was induced. Three days, two days, and one day before surgery, the mice were intraperitoneally injected with 20 mg/kg Coixol, respectively. On the day of surgery, renal vessels were clamped for 35 min, followed by 24 h of reperfusion. Finally, the mice were euthanized for subsequent experiments. (**B**) The serum creatinine levels in different groups of mice (n = 6). (**C**) The BUN levels in different groups of mice (n = 6). (**D**) Representative images and the quantification of PAS from different groups of mice (n = 6). Scale bar: 20 μm. All data are expressed as means ± SEM. * *p* < 0.05 vs. sham; # *p* < 0.05 vs. AKI.

As revealed by a machine learning analysis and RNA sequencing data validation, Plaur is the crucial target of Coixol. Next, we overexpressed Plaur by transfecting Plaur-overexpressing plasmid in AKI mice (Figure 5A). We found that Plaur overexpression significantly decreased the protective effect of Coixol on renal function, as evidenced by the reduction in SCr and BUN (Figure 5B,C). Moreover, we found that Plaur overexpression significantly impaired renal tubular damage, as evidenced by PAS staining (Figure 5D).

### 3.5. Cellular Senescence Is a Target of Plaur in Coixol-Treated AKI Mice

**Figure 6:**
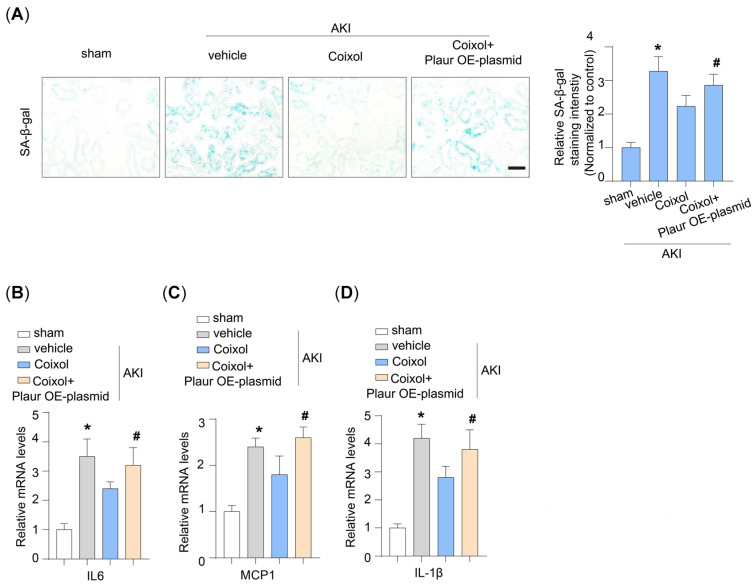
Cellular senescence is a target of Plaur in Coixol-treated AKI mice. (**A**) Representative images and the quantification of SA-β-gal staining levels in different groups of mice. Scale bar: 20 μm. (**B**) Relative mRNA levels of IL6 in different groups of mice (n = 6). (**C**) Relative mRNA levels of MCP1 in different groups of mice (n = 6). (**D**) Relative mRNA levels of IL-1β in different groups of mice (n = 6). All data are expressed as means ± SEM. * *p* < 0.05 vs. sham; # *p* < 0.05 vs. AKI.

We assessed the effect of Plaur overexpression on cellular senescence in AKI mice. It was found that Plaur overexpression significantly decreased the protective effect of Coixol on renal cellular senescence (Figure 6A). Additionally, we found that Plaur overexpression increased the SASP, as evidenced by the upregulation of IL6, MCP1, and IL-1β expression (Figure 6B–D).

### 3.6. The result of Molecular Docking

**Figure 7:**
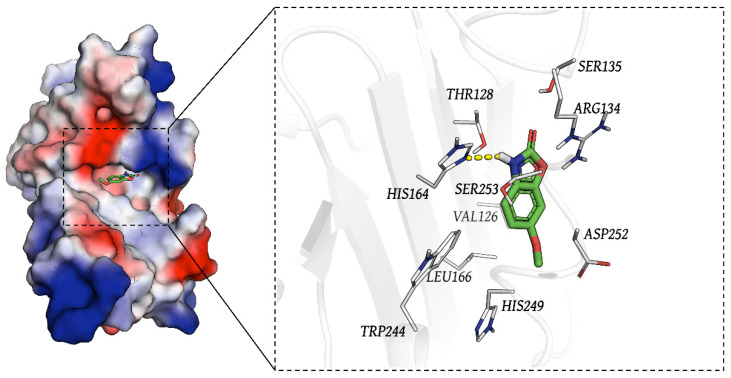
The binding mode of Plaur and Coixol. The green stick represents the small molecule, and the yellow lines indicate hydrogen bond interactions. Red and blue coloring indicate oxygen and nitrogen atoms, respectively. Coixol binds to a pocket formed by the residues TRP244, HIS249, LEU166, THR128, HIS164, ASP252, SER253, ARG134, and SER135.

Docking was conducted blindly. Figure 7 presents the interaction map between the small molecule Coixol and the Plaur protein. Coixol binds to a pocket formed by the residues TRP244, HIS249, LEU166, THR128, HIS164, ASP252, SER253, ARG134, and SER135. Additionally, the molecule forms a hydrogen bond with HIS164 on the Plaur protein. The formation of this hydrogen bond strengthens the binding between the protein and the small molecule. In this complex, the docking software provides a binding affinity score of −6.199 kcal/mol for Coixol binding to Plaur. Compared with the positive control (−5.21 kcal/mol), the binding between Plaur and Coixol was tighter, suggesting that Plaur might be a potential target for Coixol.

### 3.7. The Results of the MD Simulations

**Figure 8:**
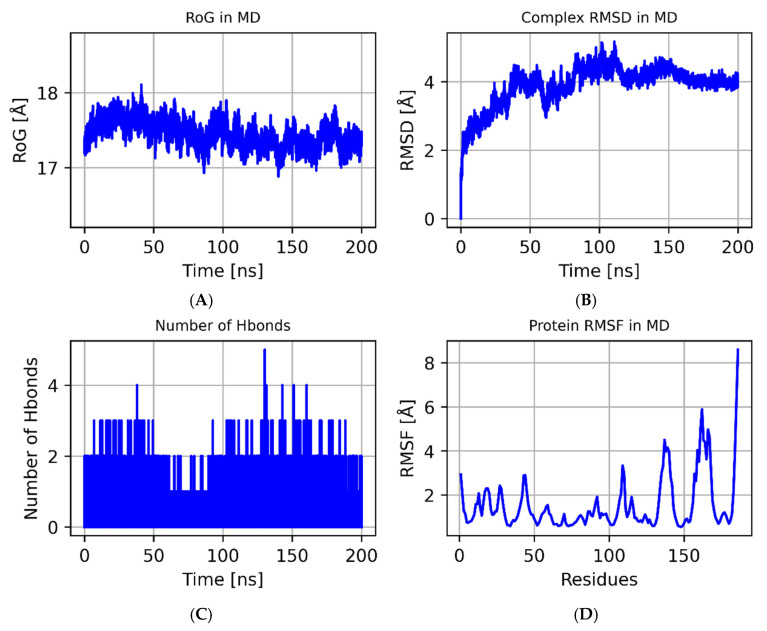
Molecular dynamics. (**A**) The RoG of the ligand–protein complex during the simulation. (**B**) The RMSD of the ligand–protein complex during the simulation. (**C**) The number of hydrogen bonds between the ligand and protein over time. (**D**) The protein’s RMSF plot.

We performed MD simulations on the Coixol–Plaur protein complex to investigate its structural stability and interactions. The variation in the radius of gyration (RoG) over time is shown in Figure 8A. During the initial phase of the simulation (0–25 ns), the RoG value gradually increased from 17.0 Å to 18.0 Å, indicating that the system underwent structural adjustments before reaching equilibrium. After 25 ns, the RoG value stabilized around 17.5 Å, suggesting that the complex converged during the latter half of the simulation and reached a higher degree of system compactness. The Root Mean Square Deviation (RMSD) values of the complex are shown in Figure 8B. The RMSD values exhibited some fluctuations during the simulation, particularly between 50 and 75 ns, where the fluctuations were more pronounced. This may be attributed to local conformational changes in the protein. Overall, the RMSD values remained within a relatively low range throughout the simulation, indicating that the overall structure of the complex remained stable. The number of hydrogen bonds over time is shown in Figure 8C. During the simulation, the number of hydrogen bonds fluctuated between one and four, indicating a dynamic hydrogen bonding interaction between Coixol and the Plaur protein. Despite these fluctuations, the number of hydrogen bonds remained relatively stable throughout the simulation, suggesting that the hydrogen bond interactions between the ligand and the protein maintained good stability during the simulation period. The Root Mean Square Fluctuation (RMSF) values of the Plaur protein are shown in Figure 8D. The RMSF values fluctuated significantly within the 50–150 residue range, indicating higher flexibility in these regions. Notably, the RMSF values significantly increased between residues 100 and 150, suggesting that these regions may have undergone significant conformational changes during the formation of the complex. Through molecular dynamics simulations, we found that the Coixol–Plaur protein complex exhibited good structural stability during the simulation. Although some structural adjustments occurred in the early stages, the complex tended to stabilize in the latter half of the simulation. A hydrogen bond analysis showed that the interactions between the ligand and the protein maintained a dynamic equilibrium. An RMSF analysis further revealed the flexibility changes in specific regions of the protein, providing valuable insights into the structural dynamics of the complex.

**Figure 9:**
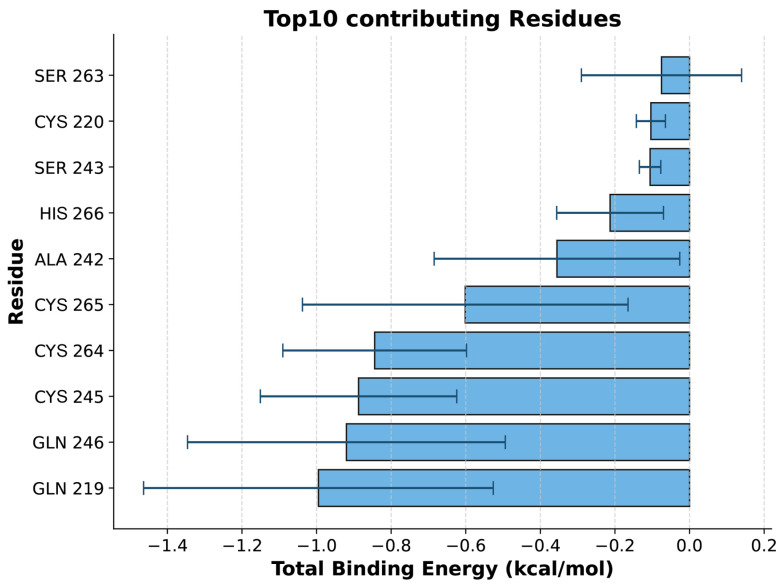
The top 10 amino acids contributing to the binding energy. GLN-219 is the key residue with the largest contribution to ligand binding.

An analysis of the binding energy contributions revealed that GLN-219 is the key residue with the largest contribution to ligand binding (Figure 9), likely stabilizing binding with Coixol through hydrophobic interactions. Additionally, multiple residues, such as GLN-246 and CYS-245, also make significant contributions to the total binding energy, indicating that these residues play a major role in forming a stable binding interface. It should be noted that molecular docking only provides a single, energy-minimized pose of the ligand within a rigid receptor model; therefore, GLN-219, GLN-246, and CYS-245 do not appear in Figure 7 because they did not contribute the strongest interactions in that static snapshot. However, over the course of the 100 ns molecular dynamics simulation, the protein–ligand complex samples a range of conformations under thermal fluctuations, and these residues repeatedly engage in hydrogen bonding and van der Waals contacts, emerging as dynamic binding hotspots. Far from contradicting the docking results, the MD simulations refine and extend the static picture by revealing additional residues whose interactions become significant under physiological motion.

### 3.8. MM-GBSA

Based on the trajectories from the molecular dynamics simulations, we calculated the binding energy using the MM-GBSA method, which more accurately reflects the binding interaction between the small molecule and the target protein. As shown in Table 1, the binding energy of Plaur_Coixol is −11.17 ± 1.76 kcal/mol. A negative value indicates a binding affinity between the two molecules and the target protein, with a lower value corresponding to stronger binding. Our calculations suggest that the binding affinity of Plaur_Coixol is very strong. Through energy decomposition, we found that the primary contributing factor to the binding of Plaur_Coixol is the van der Waals energy, followed by electrostatic energy and nonpolar solvation free energy.

## 4. Discussion

In this study, we successfully established a murine model of ischemia–reperfusion injury (IRI)-induced acute kidney injury (AKI) and demonstrated the renoprotective effects of Coixol. Through an RNA sequencing analysis, we found that activation of the cellular senescence pathway plays a pivotal role in AKI pathogenesis and that Coixol treatment effectively suppresses this activation. By integrating WGCNA and machine learning analyses, we identified Plaur as a crucial cellular senescence-related gene associated with AKI. Functional validation further showed that Plaur overexpression led to worsened renal function, increased inflammatory cytokines (IL6, MCP1, and TNF-α), and enhanced SA-β-Gal staining, suggesting that Coixol alleviates AKI partly by downregulating Plaur and inhibiting tubular cell senescence. Moreover, molecular docking and molecular dynamics simulations provided strong evidence that Plaur may serve as a direct molecular target of Coixol.

Our findings are consistent with a growing body of evidence highlighting the cellular senescence of tubular epithelial cells as a major pathological driver in chronic kidney diseases (CKDs), including diabetic kidney disease, IgA nephropathy, and hypertensive nephropathy [43,44]. Studies have shown that targeting senescent tubular cells can significantly mitigate renal damage and delay CKD progression [45]. For instance, markers such as p21, p16, and SA-β-Gal are positively associated with interstitial injury severity in IgA nephropathy, while vascular and interstitial cells exhibit enhanced senescence in hypertensive nephropathy [12]. Based on these observations, our results not only reinforce the importance of cellular senescence in renal pathologies but also suggest that Coixol, by modulating senescence pathways, may have therapeutic applications beyond AKI. Future research should explore its potential in other age-related and senescence-driven diseases, including neurodegenerative disorders, cardiovascular conditions, and tissue degenerative processes.

Additionally, this study highlights the power of machine learning in identifying novel molecular mediators in complex diseases. We found that Plaur, selected via LASSO and RF analysis, is a highly sensitive biomarker for AKI. Plaur encodes the urokinase-type plasminogen activator receptor (uPAR), which participates in extracellular matrix degradation and has been implicated in tumor metastasis, angiogenesis, and tissue remodeling [46]. Recent clinical studies have shown that elevated soluble uPAR (suPAR) levels are predictive of AKI risk [47] and mainly occur due to bone marrow-derived immature myeloid cells [48]. Mechanistically, uPAR activation can induce podocyte injury [49] and promote oxidative stress, persistent DNA damage, and cellular senescence via integrin β1 signaling [50].

Interestingly, uPAR-targeting senolytic CAR-T cells have been shown to effectively eliminate senescent cells and ameliorate senescence-associated liver diseases [51,52]. However, the role of uPAR in tubular epithelial cell senescence during AKI has not been well elucidated. Our findings provide new insights by demonstrating that Plaur upregulation contributes to tubular cell senescence in AKI and that Coixol can counteract this process by downregulating Plaur expression. Importantly, molecular docking studies further support the direct interaction between Coixol and Plaur. Coixol formed stable hydrogen bonds with Plaur and exhibited a more favorable binding energy compared to the positive control, indicating a strong and specific interaction. These results suggest that Plaur could be a potential therapeutic target of Coixol, providing a mechanistic basis for its anti-senescent effects. Gene transfer to the kidney holds great potential as a novel therapeutic approach [53,54]. In this study, we overexpressed Plaur based on high-pressure hydrodynamic injection [25]. Our study provides direct evidence that the overexpression of Plaur mitigates Coixol’s protective effects on AKI. We also observed that cellular senescence was reduced by Coixol overexpression, further indicating that Coixol may be a novel agent for senotherapeutics.

While Coixol has not yet entered clinical trials, preclinical studies in murine models have demonstrated its therapeutic potential. Notably, research indicates that Coixol is a potent, orally bioactive compound with no observed toxicity even at high doses (20–500 mg/kg/day) over extended administration periods (30 days) [11,55]. These findings highlight its promising safety profile and support its candidacy for future clinical translation.

This study has several limitations. Firstly, the use of hydrodynamic tail vein injection for plasmid delivery, although effective, presents technical challenges and may induce systemic inflammation, which could act as a confounding factor in our results. Alternative delivery methods, such as viral vectors, may offer more targeted and efficient gene transfer with fewer off-target effects. Future studies should consider employing such approaches to validate and refine our findings. Secondly, although we observed a significant increase in SA-β-Gal staining and SASP, further research is needed to clarify the mechanisms driving the accumulation of senescent cells in AKI. Thirdly, the precise molecular mechanisms by which Coixol regulates Plaur remain unclear. Our findings emphasize the role of the Plaur signaling pathway in the regulation of tubular cell senescence, but we cannot rule out the involvement of other regulatory factors in this process. Additional experiments are critical to exploring the regulatory mechanism. Fourthly, although we observed significant cellular senescence in renal tubular cells from mouse kidneys, we were unable to induce measurable senescence in cultured tubular epithelial cells under the current experimental conditions. This limitation prevented us from conducting further mechanistic studies in vitro. Future work will explore alternative stressors, prolonged exposure times, and possibly three-dimensional culture systems to better model senescence in vitro. Fifthly, our study primarily focused on the preventive effects of Coixol via pretreatment. We did not evaluate its therapeutic potential after the onset of AKI, which limits the translational relevance of our findings. Future studies are needed to investigate whether Coixol can confer renal protection when administered after injury onset, thereby better mimicking clinical treatment scenarios. Finally, the relatively small sample sizes used in this study may limit the statistical power and robustness of the findings. For example, although machine learning identified several potential target genes, not all were validated in our RNA-seq analysis. Due to the relatively small sample size, it is possible that these genes still have potential as Coixol targets. Given the inherent variability in AKI models, future studies with larger cohorts are needed to validate and strengthen these observations.

Collectively, our study confirmed for the first time that Coixol treatment significantly reduced renal injury in AKI. Particularly, Coixol ameliorated IRI-induced cellular senescence, at least in part, through regulating Plaur expression in AKI, offering a novel approach for the treatment of AKI.

## 5. Conclusions

This study demonstrates for the first time that Coixol exerts significant protective effects against acute kidney injury by reducing cellular senescence. Through comprehensive bioinformatics analyses and experimental validation, we identified Plaur as a key target mediating the protective action of Coixol. Coixol treatment not only improved kidney function and reduced tissue damage but also alleviated markers of cellular aging and inflammation. Molecular docking and dynamic simulations further confirmed a strong binding interaction between Coixol and Plaur. These findings suggest that Coixol may serve as a promising candidate for developing new therapeutic strategies targeting cellular senescence in acute kidney injury. Future studies are warranted to explore the long-term effects and therapeutic applications of Coixol in clinical settings.

## Figures and Tables

**Table 1:** Binding free energies and energy components predicted using MM-GBSA method (kcal/mol).

System Name	Plaur_Coixol
Δ*E*_vdw_	−14.10 ± 1.00
Δ*E*_elec_	−5.78 ± 5.50
ΔG_GB_	10.54 ± 4.09
ΔG_SA_	−1.83 ± 0.16
ΔG_bind_	−11.17 ± 1.76

ΔE_vdw_: van der Waals energy; ΔE_elec_: electrostatic energy; ΔG_GB_: electrostatic contribution to solvation; ΔG_SA_: nonpolar contribution to solvation; ΔG_bind_: binding free energy.

## Data Availability

The data that support the findings are available from the corresponding authors upon reasonable request.

## Supplementary Materials

The following supporting information can be downloaded at: https://www.mdpi.com/article/10.3390/biology14050560/s1, Table S1: Primer pairs of target genes used for PCR in this study; Table S2: Antibodies used in this study; Table S3: Physical and biochemical parameters of AKI mice with coixol treatment; Table S4: The results of KEGG analysis.
