# Supplementary material for: Coixol Protects Against Acute Kidney Injury by Reducing Cell Senescence
Source: Biology (Basel). 2025 May 17;14(5):560. doi: 10.3390/biology14050560 (PMC12109185; doi:10.3390/biology14050560)
Supplement: Supplementary file 1 [file biology-14-00560-s001.zip › biology-3596577-supplementary.docx]

**Table S1. Primer pairs of target genes used for PCR in this study.**

| Gene | Species | Forward (5’ to 3’) | Reverse (5’ to 3’) |
| --- | --- | --- | --- |
| IL6 | Mouse | TACCACTTCACAAGT  CGGAGGC | CTGCAAGTGCATCAT  CGTTGTTC |
| TNF-α | Mouse | TGATCGGTCCCCAAA  GGGAT | TGTCTTTGAGATCCAT  GCCGT |
| MCP1 | Mouse | GCAGCAGGTGTCCCAA AGAA | ATTTACGGGTCAACTTCA CATTCAA |
| IL-1β | Mouse | AGCTTCTCCACAGCCACAAT | TGTGAAATGCCACCTTTTGA |
| β-actin | Mouse | CATTGCTGACAGG  ATGCAGAAGG | TGCTGGAAGGTGGAC  AGTGAGG |

**Table S2. Antibodies used in this study.**

| **Primary**  **antibodies** | **Dilution and supplier** | **Product ID** | **Host** | **Application** |
| --- | --- | --- | --- | --- |
| KIM1 | 1:50; Abclonal | A28313 | Rabbit | IF |

**Table S3. Physical and biochemical parameters of AKI mice with coixol treatment.**

| **Variables** | **Normal** | **AKI** | |
| --- | --- | --- | --- |
|  | **Vehicle** | **Vehicle** | **Coixol** |
| **BW (g)** | 26.02±2.01 | 22.15±1.83 | 26.46±2.05 * |
| **KW/BW (mg/g)** | 7.21±0.64 | 9.36±0.75 | 6.53±1.26 * |
| **FBG (mmol/L)** | 4.08±0.96 | 5.38±1.04 | 4.90±1.06 |
| **Heart rate (bpm)** | 501.31±30.23 | 463.16±50.13 | 492.57±28.41 |
| **SBP (mmHg)** | 124.11±3.11 | 125.21±4.31 | 119.82±4.27 |
| **DBP (mmHg)** | 76.51±2.92 | 73.62±1.87 | 75.42±3.95 |

BW, body weight; KW/BW, kidney weight/ body weight; FBG, fasting blood glucose; SBP, Systolic blood pressure; DBP, Diastolic blood pressure.

Data are expressed as means ± SEM. **P* < 0.05 VS Vehicle AKI mice (n = 6). One-way ANOVA followed by Tukey’s post-hoc test was used.
